# Inferring Weighted Directed Association Network from Multivariate Time Series with a Synthetic Method of Partial Symbolic Transfer Entropy Spectrum and Granger Causality

**DOI:** 10.1371/journal.pone.0166084

**Published:** 2016-11-10

**Authors:** Yanzhu Hu, Huiyang Zhao, Xinbo Ai

**Affiliations:** 1 Beijing Key Laboratory of Work Safety Intelligent Monitoring, Beijing University of Posts and Telecommunications, Beijing, 100876, China; 2 School of Information Engineering, Xuchang University, Xuchang, 461000, China; Beihang University, CHINA

## Abstract

Complex network methodology is very useful for complex system explorer. However, the relationships among variables in complex system are usually not clear. Therefore, inferring association networks among variables from their observed data has been a popular research topic. We propose a synthetic method, named small-shuffle partial symbolic transfer entropy spectrum (SSPSTES), for inferring association network from multivariate time series. The method synthesizes surrogate data, partial symbolic transfer entropy (PSTE) and Granger causality. A proper threshold selection is crucial for common correlation identification methods and it is not easy for users. The proposed method can not only identify the strong correlation without selecting a threshold but also has the ability of correlation quantification, direction identification and temporal relation identification. The method can be divided into three layers, i.e. data layer, model layer and network layer. In the model layer, the method identifies all the possible pair-wise correlation. In the network layer, we introduce a filter algorithm to remove the indirect weak correlation and retain strong correlation. Finally, we build a weighted adjacency matrix, the value of each entry representing the correlation level between pair-wise variables, and then get the weighted directed association network. Two numerical simulated data from linear system and nonlinear system are illustrated to show the steps and performance of the proposed approach. The ability of the proposed method is approved by an application finally.

## Introduction

### Problem Statement

Association networks are found in many domains, such as networks of citation patterns across scientific articles [1–3], networks of actors co-starring in movies [4–6], networks of regulatory influence among genes [7, 8], and networks of functional connectivity between regions of the brain[9, 10]. The rules defining edges in association networks are not the same. In general, if the relationships among nodes are explicit, we can define a rule for their connectivity and establish the network easily. While the relationships among components are unknown in many real complex systems, so association network inference has become a popular research topic. Many complex systems belong to the industrial field and the datasets obtained from these complex systems are multivariate time series. Therefore, we aim at studying association network inference from multivariate time series and also attempt to deal with the problems of the edges’ direction and weight in the network appropriately.

### Related Works

Association network inference has been a research topic for several years. We will review some methods that have been proposed so far to addressed the undetermined relationships among variables. The most classical approach is based on correlation. For instance, Guo et al. [7] incorporated the distance correlation into inferring gene regulatory networks from the gene expression data without any underling distribution assumptions. Maucher et al. [11] used Pearson correlation as an elementary correlation measure to detect regulatory dependencies in a gene regulatory network. The association networks generated by basic correlation approach usually include many indirect relationships which need to be detected and removed to increase the power of the network inference approach. Therefore, a major challenge in inferring association networks is the identification of direct relationships between variables. The classical approach to detect indirect relationships is based on partial correlations, which imposes the control of one gene on the relationship of others. Han and Zhu [12] proposed a method based on the matrix of thresholding partial correlation coefficients (MTPCC) for network inference from expression profiles. The corresponding undirected dependency graph (UDG) was obtained as a model of the regulatory network of S. cerevisiae. Yuan et al. [8] proposed a directed partial correlation (DPC) method as an efficient and effective solution to regulatory network inference. It combines the efficiency of partial correlation for setting up network topology by testing conditional independence, and the concept of Granger causality to assess topology change with induced interruptions. Wang et al. [13] focused on gene group interactions and inferred these interactions using appropriate partial correlations between genes, that is, the conditional dependencies between genes after removing the influences of a set of other functionally related genes.

Moreover, Gaussian Graphical Models also performed well to infer association network on specific experimental dataset. Schäfer and Strimmer [14] introduced a framework for small-sample inference of graphical models from gene expression data to detect conditionally dependent genes. Huynh-Thu et al. [15] proposed an algorithm using tree-based ensemble methods Random Forests or Extra-Trees for the inference of GRNs(Genetic Regulatory Networks) that was best performer in the DREAM4 In Silico Multifactorial challenge.

Some approaches to infer association networks rely on information theoretic-based similarity measures. Margolin et al. [16] described a computational protocol for the ARACNE algorithm, an information-theoretic method for identifying transcriptional interactions between gene products using microarray expression profile data. Faith et al. [17] developed and applied the context likelihood of relatedness (CLR) algorithm, also used mutual information as a metric of similarity between the expression profiles of two genes. Zoppoli et al. [18] proposed a method called TimeDelay-ARACNE. It tries to extract dependencies between two genes at different time delays, providing a measure of these dependencies in terms of mutual information. TimeDelay-ARACNE can infer small local networks of time regulated gene-gene interactions detecting their versus and also discovering cyclic interactions when only a medium-small number of measurements are available. Villaverde et al. [19] reviewed some of the existing information theoretic methodologies for network inference, and clarify their differences.

In addition, approaches rooted in Bayesian Networks (BN) employ probabilistic graphical models in order to infer causal relationships between variables. Aliferis et al. [20] presented an algorithmic framework for learning local causal structure around target variables of interest in the form of direct causes/effects and Markov blankets applicable to very large data sets with relatively small samples. The selected feature sets can be used for causal discovery and classification. Dondelinger et al. [21] introduced a novel information sharing scheme to infer gene regulatory networks from multiple sources of gene expression data. They illustrate and test this method on a set of synthetic data, using three different measures to quantify the network reconstruction accuracy. As a review paper, Lian et al. [22] first discussed the evolution of molecular biology research from reductionism to holism. This is followed by a brief insight on various computational and statistical methods used in GRN inference before focusing on reviewing the current development and applications of DBN-based methods.

Granger causality (GC) is also a very popular tool for association networks inference. It can assess the presence of directional association between two time series of a multivariate data set. GC was introduced originally by Wiener [23], and later formalized by Granger [24] in terms of linear vector autoregressive (VAR) modeling of multivariate stochastic processes. Tilghman and Rosenbluth [25] presented Granger Causality as a method for inferring communications links among a collection of wireless transmitters from externally measurable features. The link inference method was applicable to inferring the link topology of broad classes of wireless networks, regardless of the nature of the Medium Access Control (MAC) protocol used. Cecchi et al. [9] presented a scalable method, based on the Granger causality analysis of multivariate linear models, to compute the structure of causal links over large scale dynamical systems that achieves high efficiency in discovering actual functional connections. The method was proved well to deal with autoregressive models of more than 10,000 variables. Schiatti et al. [26] compared the GC with a novel measure, termed extended GC (eGC), able to capture instantaneous causal relationships. The practical estimation of eGC worked with a two-step procedure, first detecting the existence of zero-lag correlations, and then assigning them to one of the two possible causal directions using pairwise measures of non-Gaussianity.

Of course, there are many more methods for association networks inference and we have not mentioned above, such as neural network [27], SparCC [28], S estimator [29, 30], Maximal Information Coefficient (MIC) [31], Local Similarity Analysis (LSA) [32, 33], and so on. They all showed some excellent performance through experiment and observation.

Although any of the abovementioned researches have its advantages approved by different styles, it is not always suitable for any network inference problem. Because each strategy applies different assumptions, they each have different strengths and limitations and highlight complementary aspects of the network. In this paper, we aim at inferring weighted directed association network from multivariate time series and the abovementioned methods can’t meet our requirements well. For instance, some of these popular tools are non-directional, e.g. correlation or partial correlation, mutual information measures and Bayesian Networks, thus these measures cannot satisfy one’s directed association networks inference study [34]. Granger causality is able to detect asymmetry in the interaction. However, its limitation is that the model should be appropriately matched to the underlying dynamics of the examined system, otherwise model misspecification may lead to spurious causalities [35]. Some of the proposed methods cannot detect indirect relationships, such as basic correlation, mutual information and Bayesian Networks. Some of the proposed methods mainly deal with linear problem, e.g. Pearson correlation and Spearman correlation, but are not appropriate for nonlinear problem.

### Primary Contribution of This Work

To address the issues mentioned above, we will propose an approach called small-shuffle partial symbolic transfer entropy spectrum(SSPSTES). This work face with five challenges:

1. Time series being non-stationary and continuous: It is very important that the time series is statistically stationary over the period of interest, which can be a practical problem with transfer entropy calculations [36]. In addition, it is problematic to calculate the transfer entropy on continuous-valued time series. Thus, here we will resort to an extended solution of transfer entropy, i.e. symbolic transfer entropy.
2. Threshold selection: Many current methods, e.g. correlation efficient, mutual information and transfer entropy, decide whether exists an edge between two time series by threshold selection. If a larger value is selected, it will loss many real correlations and result a sparse network. By contrast, if a smaller threshold is selected, it will bring many spurious relationships and result a dense network. Although there are many researches on threshold selection, it is still difficult for user to select a proper threshold when inferring association network. The proposed method is a solution for this problem.
3. Strong relationships identification: In general, we are more interested in the strong correlation than weak correlation. Because the relationships among these variables are unknown, strong correlations are more convincing but weak correlations have a greater probability of misidentification and this may bring a serious consequence. In addition, strong correlation is usually direct relation and not indirect relation. It is expected in the inference of association network.
4. The direction and quantity of influence: The direction of edge is crucial for network prediction and evolution. It means that the proposed method should have the ability of detecting the directional influence that one variable exerts on another between two variables.
5. Temporal relation identification: The proposed method has some ability of detecting the specific temporal relation based time lags, namely the function relation of time.
6. In the next section, we will propose a method of inferring association network from multivariate time series. The emphasis is on how to solve the five challenges mentioned above. Section 3 will apply the proposed method to two numerical examples whose coupled relationships of their components are clear and the values are time-varying. We summarize the results of this paper and figure out some topics for further study in Section 4.

## Methods

In this section, we will explain the proposed approach in detail. First, we will show you an integrated framework of the approach, and then carry out a detailed description around the framework.

### Main Principle

**Fig 1:**
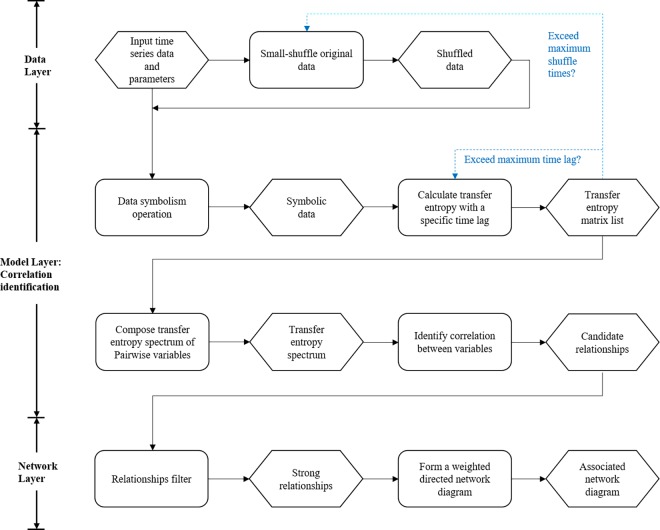
Transfer Entropy-based framework to infer association networks from multivariate time series. The black solid arrowed line in the flow diagram represents the determined sequential process and the blue dashed arrowed line, along with a Boolean condition, represents potential process. When the value of condition expression is false, the corresponding process will be carried out. Each rounded rectangle represents a key processing operations using a specific method and each hexagon represents a staged result.

The approach designed for association network inference takes exploration and application into account so that minimizing human intervention when modeling. Therefore, the approach starts with inputting data and ends with outputting a network inferred from multivariate time series. The modelling process is transparent for users. The main principle of the proposed approach is shown in Fig 1.

The integrated framework has three layers. The first layer, so-called Data Layer, is the interface interaction with users. One thing to do in this layer is to input the original multivariate time series and modelling parameters, the other thing to do is to shuffle the original data several times with a surrogate data method. The most important and complicated layer of the framework is the second layer, i.e. Model layer. We will identify all the impossible relationships among the multivariate time series in this layer. In order to achieve this goal, the core things to do are time series symbolism, partial symbolic transfer entropy calculation and spectrum construction. The output of this layer is candidate relationships. The task of last layer is to construct a weighted directed network. In order to retain the strong correlation only, the candidate relationships are filtered. For the indirect correlation, it is removed by DPI(Data Processing Inequality)[37]. For the bidirectional correlation, we deal with this problem by an empirical criterion. In the inferred association network, the start node of an arrowed edge represents a driven variable and the end node represents its corresponding variable. The weight of an edge quantifies the correlation between two nodes, i.e. time series variables.

As shown in Fig 1, there are seven key processing operations, represented by rounded rectangles, to accomplish association networks inference. Thus, we will introduce the seven steps one by one in the rest of this section.

### Small-Shuffle Surrogate Data Method

The technique of surrogate data analysis is a randomization test method [38]. Given time series data, surrogate time series are constructed consistent with the original data and some null hypothesis. The random-shuffle surrogate (RSS) method proposed in [38] can test whether data can be fully described by independent and identically distributed random variables. As summarized in [38, 39], the limit of RSS method is that it destroys any correlation structure in data. That is, not only the short-term relationship but also the long-trend relationship between two variables are also destroyed. The RSS method assumes global stationarity and performs a pairwise linear decoupling between channels. But in many typical examples the individual channels are also influenced by other nonstationary variation. So we prefer to use the small-shuffled surrogate (SSS) method proposed in [39–41].

The SSS method destroys local structures or correlations in irregular fluctuations (short-term variabilities) and preserves the global behaviors by shuffling the data index on a small scale. The steps using SSS method are described as follows.

Let the original data be *x*(*t*), let *i*(*t*) be the index of *x*(*t*) [that is, *i*(*t*) = *t*, and so *x*(*i*(*t*)) = *x*(*t*)], let *g*(*t*) be Gaussian random numbers, and *s*(*t*) will be the surrogate data.

- Shuffle the index of *x*(*t*):
  $$i^{'}(t)=i(t)+A\times g(t)$$ (1)
  where A is an amplitude.
- Sort *i*'(*t*) by the rank order and let the index of *i*'(*t*) be $\hat{i}(t)$.
- Obtain the surrogate data:

$$s(t)=x[\hat{i}(t)]$$ (2)

Parameter A reflect the extent of shuffling data. A higher value of parameter A results more difference between surrogate data and original data. On the contrary, the smaller the value of A, the less the difference. The parameter A is input at the beginning of the method and its empirical value of A is 1.0.

### Time Series Symbolization

The technique of time series symbolization was introduced with the concept of permutation entropy [42, 43]. This technique makes many other researches on time series get new progress and bring us some new techniques, e.g. permutation entropy [42] and symbolic transfer entropy(STE) [43]. It is helpful to deal with the problem of continuous and non-linear time series. The principle of time series symbolization is described as follows:

For original multivariate time series, let two time series *V*_1_,*V*_2_, be {*v*_1,*t*_}, {*v*_2,*t*_} respectively, *t* = 1,2,⋯,*k*. The embedding parameters in order to form the reconstructed vector of the time series *V*_1_ are the embedding dimension *m*_1_ and the time delay *τ*_1_. Accordingly, *m*_2_ and *τ*_2_ are the embedding parameters defined for *V*_2_. The reconstructed vector of *V*_1_ is defined as:

$$\nu_{1,t}={\left(v_{1,t},v_{1,t-\tau_{1}},\cdots ,v_{1,t-(m_{1}-1)\tau_{1}}\right)}^{'},$$ (3)

where *t* = 1,2,⋯,*k*' and *k*' = *k* − max((*m*_1_−1)*τ*_1_,(*m*_2_−1)*τ*_2_).

For each vector **ν**_1,*t*_, the ranks of its components assign a rank-point ${\hat{\nu }}_{1,t}=[r_{1,t},r_{2,t},\cdots ,r_{m_{1},t}]$ where *r*_*j*,*t*_ ∈ {1,2,⋯,*m*_1_} for *j* = 1,2,⋯,*m*_1_. ${\hat{\nu }}_{2,t}$ is defined accordingly.

### Partial Symbolic Transfer Entropy Calculation with Different Time Lags

Symbolic transfer entropy means that our transfer entropy calculation is based on symbolic time series data in section 2.3. Symbolic transfer entropy is defined as follows [43]:

$$STE_{v_{2}\to v_{1}}=\sum p({\hat{\nu }}_{1,t+\tau },{\hat{\nu }}_{1,t},{\hat{\nu }}_{2,t})\log\frac{p({\hat{\nu }}_{1,t+\tau }|{\hat{\nu }}_{1,t},{\hat{\nu }}_{2,t})}{p({\hat{\nu }}_{1,t+\tau }|{\hat{\nu }}_{1,t})},$$ (4)

where *τ* is the time delay, $p({\hat{\nu }}_{1,t+\tau },{\hat{\nu }}_{1,t},{\hat{\nu }}_{2,t})$, $p({\hat{\nu }}_{1,t+\tau }|{\hat{\nu }}_{1,t},{\hat{\nu }}_{2,t})$ and $p({\hat{\nu }}_{1,t+\tau }|{\hat{\nu }}_{1,t})$ are the joint and conditional distributions estimated on the rank vectors as relative frequencies, respectively.

Symbolic transfer entropy uses a convenient rank transform to find an estimate of the transfer entropy on continuous data without the need for kernel density estimation. Since slow drifts do not have a direct effect on the ranks, it still works well for non-stationary time series [34].

The partial symbolic transfer entropy(PSTE)[34] is defined conditioning on the set of the remaining time series *z* = {*v*_3_,*v*_4_,⋯,*v*_*n*_}.

$$PSTE_{v_{2}\to v_{1}}=\sum p({\hat{v}}_{1,t+\tau },{\hat{v}}_{1,t},{\hat{v}}_{2,t},{\hat{z}}_{t})\log\frac{p({\hat{v}}_{1,t+\tau }|{\hat{v}}_{1,t},{\hat{v}}_{2,t},{\hat{z}}_{t})}{p({\hat{v}}_{1,t+\tau }|{\hat{v}}_{1,t},{\hat{z}}_{t})},$$ (5)

where the rank vector ${\hat{z}}_{t}$ is defined as the concatenation of the rank vectors for each of the embedding vectors of the time series in *z*. The partial symbolic transfer entropy is similar as partial correlation, it can eliminate some of the indirect correlation and remain the pure or direct information flow between *v*_2_ and *v*_1_.

Due to the time delay is underdetermined, the partial symbolic transfer entropy is calculated *n* times for each pair of time series. This process is described using algorithm 1 shown in Box 1.

**Box 1. The process of calculating partial symbolic transfer entropy with different lags**

**Algorithm 1**: **Partial Symbolic Transfer Entropy Calculation with Different Time Lags**

**Input:** *tm*, maximum time delay

**Output:** *PSTEML*, a list of partial symbolic transfer entropy matrix

**Method**:

for (t = 1; t< = *tm*; t++) {

    colNum = column number of time series data

    for (i = 1; i< = colNum; j++) {

        for (j = 1; j< = colNum; j++) {

                if (j ≠ i) {

                    STS = call the function of time series symbolization

                STS(j) = the column j of STS

                STS(i) = the column i of STS

                STS(z) = the columns z of STS

                PSTE_matrix [i, j] = call_PSTE_Function(STS(j), STS(i), STS(z), t)

            }

        }

    }

    Element t of *PSTEML* = PSTE_matrix

}

**Return** *PSTEML*

We first use algorithm 1 to get a list of symbolic transfer entropy matrix on original time series. Then we shuffle the original data several times which has been specified at the beginning of our method. We repeat the algorithm 1 on each shuffled data accordingly.

### Partial Symbolic Transfer Entropy Spectrum Composition

Partial Symbolic Transfer Entropy Spectrum(PSTES) is defined as follows:

The PSTES between time series Y and X is composed of their many partial symbolic transfer entropy curves drawn in a rectangular coordinate system. The horizontal axis represents different time delays and the vertical axis represents transfer entropy. One of the transfer entropy curves is resulted from original data and other curves are resulted from shuffled data.

Let $L_{Y\to X}^{o}$ be the transfer entropy curve of original data, $L_{Y\to X}^{s}$ be the transfer entropy curve of shuffled data, then PSTES between Y and X can be denoted as follows:

$$PSTES_{Y\to X}=\{L_{Y\to X}^{o},\{L_{Y\to X}^{s}\}\}$$ (6)

In order to compose transfer entropy spectrum, we must understand the structure of the output in section 2.4. The output is a complicated list of PSTE matrix. For each data, original data or shuffled data, a list of PSTE matrix with different delays is returned after carrying out algorithm 1. Thus, for all data, the returned result of last step is a list of PSTE matrix lists. The parameters input at the beginning of the method are maximum time delay *tm* and shuffling times *sm*. Let *tm* = 10, *sm* = 99, then the output of last step is a list of 100 elements and each element is a list of 10 transfer entropy matrices. Moreover, each entry of the transfer entropy matrix reflects the correlation strength of a pair of time series. Thus, according to the define of PSTES, we first split the output of section 2.4 into pieces and then compose partial symbolic transfer entropy spectrum in a certain way.

### Correlation Identification and Filter

#### Candidate relationships identification

The target of the proposed method in this paper is strong correlation identification and is not all correlation among multivariate time series. The scenario for this method is that we don’t know the relationships in the complex system. We pay more attention to the precision of correlation identification than the sensitivity. Because the misidentification of relationships among variables may bring a serious consequence to our data analysis.

Our decision whether existing a strong correlation or not between two variables is made by the characteristic of PSTES. This characteristic is based on the theory of hypothesis testing which is often used in surrogate data method [30, 34, 38, 41]. Discriminating statistics are necessary for surrogate data hypothesis testing. The cross correlation and average mutual information were selected as discriminating statistics in [40, 41], and partial symbolic transfer entropy in [34]. In this paper, we consider transfer entropy as discriminating statistics. The surrogate data method also need a null hypothesis. Applying a statistical hypothesis test can result in two outcomes, i.e. the null hypothesis is rejected or not. There are two type of errors when using the hypothesis testing. If the null hypothesis is rejected and it is true, this is called type I error; if we fail to reject the null hypothesis when it is in fact false, this is called type II error. The null hypothesis in our proposed method is that there is no short-term correlation structure between the data or that the irregular fluctuations are independent. In the symbolic transfer entropy spectrum, if the symbolic transfer entropy of the original data falls outside the distribution of the SSS data and existing an outlier point that its value is greater than any other points’ value, we can reject the null hypothesis. As a result, we consider that there is a short-term correlation structure between the data and this correlation is a strong correlation. Otherwise, we accept the null hypothesis and consider that there is not a strong correlation between the data. The output of this step is an adjacency matrix and its entry *a*_*ij*_ is denoted as follows:

$${\text{a}}_{ij}=\{\begin{array}{l} 1,\exists PSTE_{i\to j}^{o}(t)>\max(PSTE_{i\to j}^{s}(t))\\ 0,others \end{array}$$ (7)

where *t* ∈ (1,2,⋯,*tm*), *s* ∈ (1,2,⋯,*sm*), $PSTE_{i\to j}^{o}(t)$ is the partial symbolic transfer entropy from variable *i* to variable *j* with a time delay *t* based on the original data and $PSTE_{i\to j}^{s}$ is the partial symbolic transfer entropy with all different time delays from variable *i* to variable *j* based on the shuffled data.

#### Relationships Filter

In order to retain the strong correlation only, the candidate relationships are filtered. In order to deal with the indirect correlation, three ideas are synthesized into the filter method.

The first component of the filter method is DPI(Data Processing Inequality)[37]. The data processing inequality of information theory states that given random variables *X*, *Y* and *Z* which form a Markov chain in the order *X—*>*Y*—>*Z*, then the mutual information between *X* and *Y* is greater than or equal to the mutual information between X and Z. Of course, the mutual information between *Y* and *Z* is greater than or equal to the mutual information between X and Z. PSTE is extended from mutual information, so we deal with indirect relations according to the following equations:

IF *PSTE*_*X*→*Z*_ ≤ *PSTE*_*X*→*Y*_ and *PSTE*_*X*→*Z*_ ≤ *PSTE*_*Y*→*Z*_,

THEN the relationship between X and Z is removed.

Second, for the bidirectional correlation, we deal with this problem by an empirical criterion. The criterion is defined as follows:

IF *PSTE*[*i*,*j*]*0.4 >= *PSTE*[*j*,*i*], THEN *PSTE*[*j*,*i*] = 0.

IF *PSTE*[*j*,*i*]*0.4 >= *PSTE*[*i*,*j*], THEN *PSTE*[*i*,*j*] = 0.

Third, although PSTE measures the correlation of variation trend, it doesn’t measure the correlation of value. As a complementary method, we introduce Granger causality which is based on the residual of linear model. The strategy is as follows:

IF *GC*[*i*,*j*] = 0, THEN *PSTE*[*i*,*j*] = 0.

After this step, we will get the final 0–1 adjacency matrix. If *a*_*ij*_ = 1, the relationship between *i* and *j* is called strong relationship.

### Association Network Inference

The association network inferred from multivariate time series can be denoted as *G* = (*V*,*E*). Here *V* = {*v*_1_,*v*_2_,⋯,*v*_*n*_} is the set of vertices, i.e. time series variables, and *E* is the set of edges, i.e. the strong correlations, identified in the section 2.6, between each pair of vertices in *V*.

From the 0–1 adjacency matrix from the last step, we have determined the direction of the network. In this step, we assign a weight to the edges in *E*. The selected measure for the weight is the corresponding maximum symbolic transfer entropy of original data calculated in section 2.4 and the Eq (6) is transformed as follows:

$$a_{ij}{}^{\prime }=\{\begin{array}{l} \max(STE_{i\to j}^{o}(t)),\;a_{ij}=1\\ \;0,\;a_{ij}=0 \end{array}$$ (8)

where *i* is the driven variable, and *j* is the response variable. Finally, we can plot the association network based the weighted adjacency matrix denoted as Eq (7) and carry out deep network analysis.

## Results

In this section, we demonstrate the application of the propose method to simulated time series data from two types of complex system, i.e. linear system and nonlinear system. The relationships among the variables in these two examples is clear and therefore we can assess our method by some measures.

In all the following cases, the parameters for modelling with SSSTES method are shuffling amplitude *A* = 1.0, the dimension of symbolic time series *m* = 3, maximum time delay *tm* = 10, maximum shuffling times *sm* = 99, time point *t* = 1,2,⋯,1000. These parameters are input in the Data Layer shown in Fig 1.

### Numerical Example from linear system

**Fig 2:**
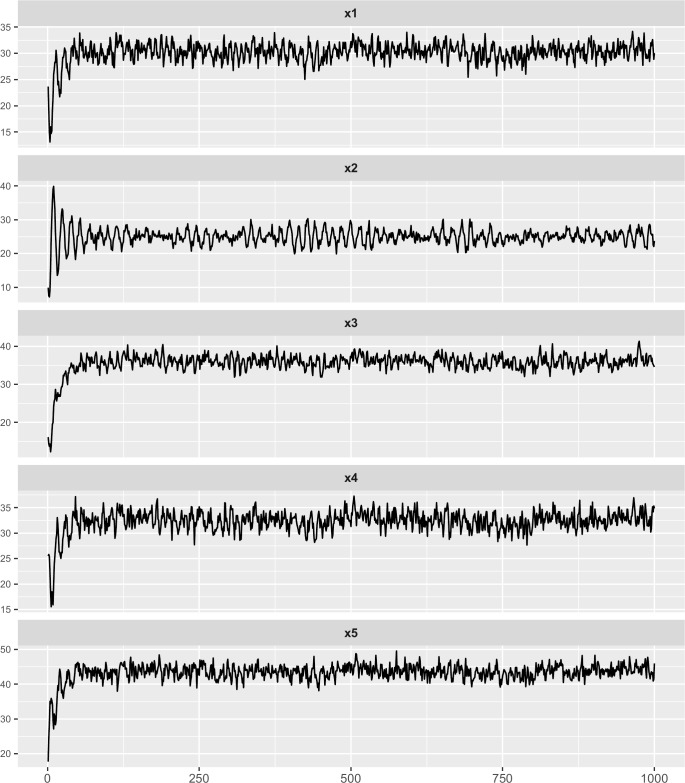
Linear multivariate time series generated by Eqs (9)–(13). This figure shows the five time series of variables *x*_1_(*t*), *x*_2_(*t*), *x*_3_(*t*), *x*_4_(*t*), *x*_5_(*t*) with titles x1, x2, x3, x4, x5.

First, we apply our method to a linear system which has five time series variables, i.e. *x*_1_(*t*), *x*_2_(*t*), *x*_3_(*t*), *x*_4_(*t*), *x*_5_(*t*). The relationships among these variables are modelled by the following expressions [41]:

$$x_{1}(t)=1.3+0.2x_{1}(t-1)+0.4x_{2}(t-4)+0.4x_{4}(t-7)+r_{1}(t),$$ (9)

$$x_{2}(t)=20+0.6x_{2}(t-1)-0.4x_{2}(t-6)+r_{2}(t),$$ (10)

$$x_{3}(t)=2.2+0.2x_{1}(t-2)+0.5x_{3}(t-1)+0.3x_{4}(t-9)+r_{3}(t),$$ (11)

$$x_{4}(t)=1.5+0.7x_{1}(t-2)+0.3x_{4}(t-1)+r_{4}(t),$$ (12)

$$x_{5}(t)=10+0.9x_{4}(t\;\text{-}\;4)+0.1x_{5}(t\;\text{-}\;1)+r_{5}(t).$$ (13)

where *r*_*i*_(*t*)(*i* = 1,2,3,4,5) are random noise, independent and identically distributed Gaussian random variables with mean zero and standard deviation 1.0. These five time series are shown in Fig 2.

**Fig 3:**
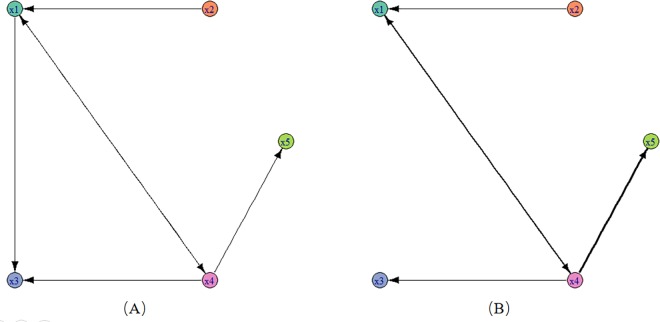
The original network and inferred networks. (A) the original association network constructed from Eqs (9)–(13); (B) the inferred association network in section 3.1.

It is difficult for us to find the relationships among the five time series variable from Fig 2. Their fluctuations seem to be irregular and don’t have obvious trend but they have linear relationships in real. If the variable *y* is a linear combination of variables *x*_1_,*x*_2_,⋯,*x*_*n*_, we say *y* is a response variable and *x*_1_,*x*_2_,⋯,*x*_*n*_ are the drive variables. In the network, we denote the drive-response relationship between *y* and *x*_1_ as a arrowed edge from *x*_1_ to *y*. Therefore, the responding network of above linear system is shown in Fig 3(A).

As shown in Fig 3(A), variable *x*_1_ is driven by two other variables *x*_2_,*x*_4_, variable *x*_3_ is driven by variables *x*_1_,*x*_4_ and *x*_4_ is driven by *x*_1_. However, *x*_2_ and *x*_5_ is not driven by any other variables and it is only as a driven variable of *x*_1_. It is noted that there are autocorrelations in Eqs (9)–(13) but we do not show the autocorrelations in Fig 3(A). In this paper, we focus on the relationships among different variables but not concern the autocorrelation.

**Fig 4:**
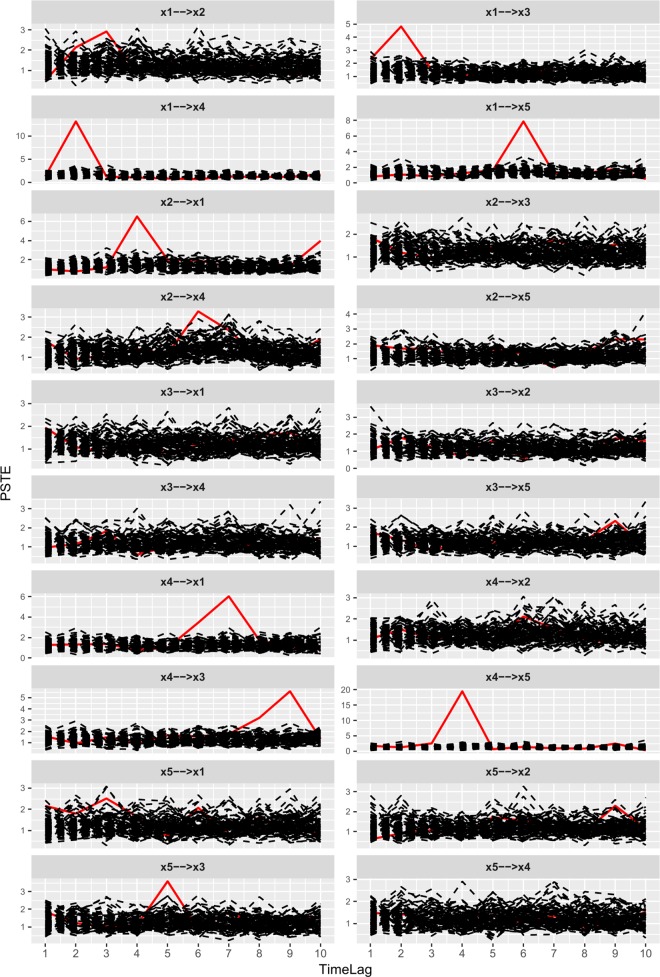
Partial symbolic transfer entropy spectrums of linear system. Plot x1—>x2 is the partial symbolic transfer entropy spectrum between time series *x*_1_ and *x*_2_. Plot x1—>x3 is the partial symbolic transfer entropy spectrum between time series *x*_1_ and *x*_3_. Other plots represent the corresponding PSTES.

After generating the simulated data(S1 Dataset) by Eqs (9)–(13) in Data Layer shown in Fig 1, what we should do is modelling with the proposed method SSPSTES. This process has been described in detail in section 2.3, 2.4, 2.5, 2.6. The shuffled data used in modelling process is generated with the method described in section 2.2. One output of the Model Layer is the symbolic transfer entropy spectrums shown in Fig 4. Since the PSTE values are rather small, they are multiplied by 100 for ease of plotting. There are twenty pairs of relationships among five time series and they are all shown in Fig 4. Per line have two pairs of relationships. The horizontal axis represents different time delays and the vertical axis represents partial symbolic transfer entropy. In each plot, the red curve is resulted from original data and other curves are resulted from shuffled data.

Then, we need to identify the candidate relationships from Fig 4. The method to identify the candidate relationships is described in section 2.6.1. This method can be described in an easy way whether part of the red curve stands outside the black curves. With this method, ten pairs of relationships are identified as candidate relationships, i.e. x1—>x3, x1—>x4, x2—>x1, x4—>x1, x4—>x3, x4—>x5, x5—>x3, x1—>x2, x1—>x5, x2—>x4. By contrast with Eqs (9)–(13), we find that first six identified relationships are correct and the others are redundant.

Next, the candidate relationships are filtered by the method described in section 2.6.2. After this step, we get all the strong relationships and the output is a 0–1 adjacency matrix. The resulted adjacency matrix is described by Eq (14):

$$C=\left(\begin{array}{ccccc} 0 & 0 & 0 & 1 & 0\\ 1 & 0 & 0 & 0 & 0\\ 0 & 0 & 0 & 0 & 0\\ 1 & 0 & 1 & 0 & 1\\ 0 & 0 & 0 & 0 & 0 \end{array}\right)$$ (14)

From this adjacency matrix, we find that five candidate relationships are removed and the other five retained relationships are considered as strong relationships, i.e. x1—>x4, x2—>x1, x4—>x1, x4—>x3, x4—>x5. These identified strong relationships are all correct but one real relationship is filtered out mistakenly, i.e. x1—>x3.

Finally, we infer a weighted directed association network in the last layer. From Eq (14), we can get a directed network and then we should quantify the correlation strength between those pairs of relationships that have been identified out above. Therefore, we introduce a correlation measure into adjacency matrix *C* and get a new weighted adjacency matrix *C*′, whose entries is described as Eq (8). The selected measure is the maximum partial symbolic transfer entropy with different time lags of original data. Then, we get the weighted adjacency matrix *C*′ as follows:

$$C^{\prime }=\left(\begin{array}{ccccc} 0 & 0 & 0 & 13.15 & 0\\ 6.53 & 0 & 0 & 0 & 0\\ 0 & 0 & 0 & 0 & 0\\ 6.03 & 0 & 5.58 & 0 & 19.49\\ 0 & 0 & 0 & 0 & 0 \end{array}\right)$$ (15)

The association network corresponding to the matrix *C*′ is shown in Fig 3(B). In Fig 3(B), each time series is mapped as a node, and each arrowed edge stands for a drive-response relationship, and we associate each edge with a weight value, i.e., the max partial symbolic transfer entropy value, which is mapped as the width of the lines. As we see, the relationship from *x*_4_ to *x*_5_ is the most strongest one. In Fig 3, the original network (A) has six directed edges and the inferred network (B) has five edges. By comparison, we find that the five edges of inferred network all exist in the original network, thus we get a higher precision.

In order to assess the performance of the proposed method, we use two indicators, i.e. precision and sensitivity(or recall, true positive rate) [44, 45]. Precision is defined as Eq (16) and sensitivity is defined as Eq (17).

$$P=\frac{TP}{TP+FP},$$ (16)

$$S=\frac{TP}{TP+FN}.$$ (17)

Here, *TP* is the numbers of edges which are in the intersection between original edge set and inferred edge set, *FP* is the number of edges which is in inferred edge set but not in original edge set and *FN* is the number of edges which is not in inferred edge set but in the original edge set. In order to test whether the model is sensitive to the system noise, we generate ten groups of data generated by *Eqs (9)–(13)* and then apply the proposed method to these data. As a result, we get ten precision values and sensitivity values and their average values shown in Table 1. From Table 1, the average precision of our model is higher to 0.86 and the average sensitivity achieve to 0.80 although it is inferior to precision.

**Table 1:** The model assessment on 10 groups of data generated by Eqs (9)–(1,3). The values of Precision, Sensitivity and PTL in the table are rounded to two decimals.

ID	Precision	Sensitivity	PTL
1	0.83	0.83	1.00
2	0.83	0.83	1.00
3	1.00	0.67	1.00
4	0.80	0.67	1.00
5	0.83	0.83	1.00
6	0.83	0.83	1.00
7	1.00	0.83	1.00
8	0.83	0.83	1.00
9	0.83	0.83	1.00
10	0.83	0.83	1.00
Average	0.86	0.80	1.00

Next, we discuss the temporal relation identification of the proposed method. Please note that the following discussion is based on those edges inferred correctly. The time lag assigned to two correlation variables is the time point when PSTE of original data achieve the maximum value. Based on this definition, we define a measure, i.e. the precision of time lags(PTL), to assess the temporal relation identification of the proposed method. It is defined as Eq (18):

$$PTL=\frac{TPL}{TPL+FPL}.$$ (18)

Here, *TPL* is the correct number of temporal relation identification in those edges which have been identified correctly, *FPL* is the error number of temporal relation identification in those edges which have been identified correctly. The results of *PTL* are shown in Table 1. We get a higher PTL 1.00.

In addition, we discuss how the dimension of symbolic time series affects the performance of the proposed method and the results are shown in Table 2. With dimension 2, the precision is 0.84 and the sensitivity is 0.70. With dimension 3, the precision is 0.86 and the sensitivity is 0.80.

**Table 2:** The model assessment on different dimensions of symbolic time series from linear system. The values of Precision and Sensitivity in the table are rounded to two decimals.

ID	Dimension	Precision	Sensitivity
1	2	0.84	0.70
2	3	0.86	0.80

We also discuss how the length of data affects the performance of the proposed method and the results are shown in Table 3. It is found that the precision is more higher with the increase of the length of data. The sensitivity is unstable, but it keeps a high level. Although the performance of the proposed method is affected by the data length, we still get a good result when the length of data is small such as 500.

**Table 3:** The model assessment on different lengths of data from linear system. The values of Precision and Sensitivity in the table are rounded to two decimals.

ID	DataLength	Precision	Sensitivity
1	500	0.80	0.67
2	1000	0.86	0.80
3	2000	0.93	0.70
4	3000	0.97	0.90

SSPSTES is a synthetic method, we make a comparison between the proposed method and some other common methods. The results are shown in Table 4. The precision of SSPSTES is highest, i.e. 0.86. The sensitivity of SSPSTES is higher than two other methods, i.e. STE and PSTE. Although the sensitivity of GC [24, 46] is highest, its precision is too small. Therefore, we conclude that SSPSTES is good at inferring association network from linear time series. The selected p value of GC is 0.01. The selected threshold value of STE and PSTE is the mean value. If the STE or PSTE between two time series variables is bigger than the mean value, we say there is a strong relationship between these two variables.

**Table 4:** The performances of different methods on the same data from linear system. The values of Precision and Sensitivity in the table are rounded to two decimals.

ID	Method	Precision	Sensitivity
1	GC	0.41	1.00
2	STE	0.79	0.70
3	PSTE	0.78	0.77
4	SSPSTES	0.86	0.80

### Numerical Example from nonlinear system

In this section, we validate whether the proposed method work well for nonlinear system. The simulated data is generated by Eqs (19)–(24):

$$x_{1}(t)=2.7+0.5x_{1}(t\;\text{-}\;1)+r_{1}(t),$$ (19)

$$x_{2}(t)=1.7+0.2x_{2}(t-1)+0.3x_{1}^{2}(t-1)+r_{2}(t),$$ (20)

$$x_{3}(t)=1.4+0.15x_{3}(t-1)+0.8\sqrt{x_{1}(t-3)}+r_{3}(t),$$ (21)

$$x_{4}(t)=2.1+0.25x_{4}(t-1)-0.7x_{5}(t-2)+0.6x_{3}(t-4)+r_{4}(t),$$ (22)

$$x_{5}(t)=1.5+0.35x_{5}(t-1)-0.5x_{4}(t-3)+r_{5}(t),$$ (23)

$$x_{6}(t)=1.3+0.2x_{6}(t-1)+0.4x_{2}(t-1)x_{3}(\text{t}-5)+r_{6}(t).$$ (24)

**Fig 5:**
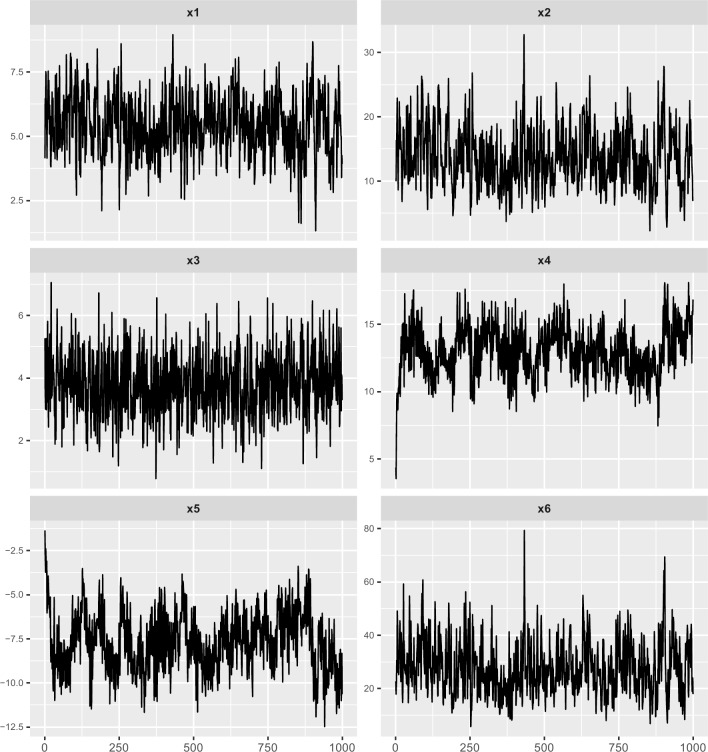
Nonlinear multivariate time series generated by Eqs (19)–(24). This figure shows the time series of variables *x*_1_(*t*), *x*_2_(*t*), *x*_3_(*t*), *x*_4_(*t*), *x*_5_(*t*), *x*_6_(*t*) with titles x1, x2, x3, x4, x5, x6.

Here, *r*_*i*_(*t*)(*i* = 1,2,3,4,5,6) are random noise, independent and identically distributed Gaussian random variables with mean zero and standard deviation 1.0. In this example, all variables except *x*_1_ are nonlinear. In Eq (20), there is a square item $x_{1}^{2}(t-1)$ and this results that *x*_2_ is nonlinear. In Eq (21), there is a square root item $\sqrt{x_{1}(t-3)}$ and this results that *x*_3_ is nonlinear. In Eq (22), there is a product 0.6*x*_3_(*t*−4) and this results that *x*_4_ is nonlinear. In Eq (23), there is a product 0.5*x*_4_(*t*−3) and this results that *x*_5_ is nonlinear. In Eq (24), there is a product 0.4*x*_2_(*t*−1)*x*_3_(*t*−5) and this results that *x*_6_ is nonlinear. In this example, we introduce into three kinds of direct nonlinear correlations, i.e. square correlation, square root correlation and the product of two one-order item. The time series(S2 Dataset) generated by Eqs (19)–(24) are shown in Fig 5.

**Fig 6:**
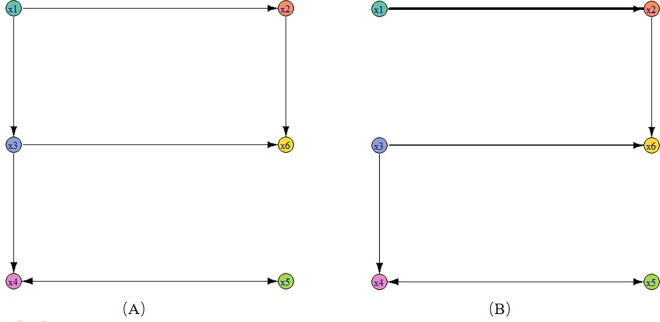
The original network and inferred networks. (A) the original association network constructed from Eqs (19)–(24); (B) the inferred association network in section 3.2.

According to the drive-response relationships among the six time series variables, the responding original network of this nonlinear system is shown in Fig 6(A). In this Fig, we can see three kinds of nodes. The first kind of nodes is that the out-degree is zero, e.g. *x*_1_. The second kind of nodes is that the in-degree is zero and the third kind of nodes is that both the out-degree and the in-degree are not zero.

**Fig 7:**
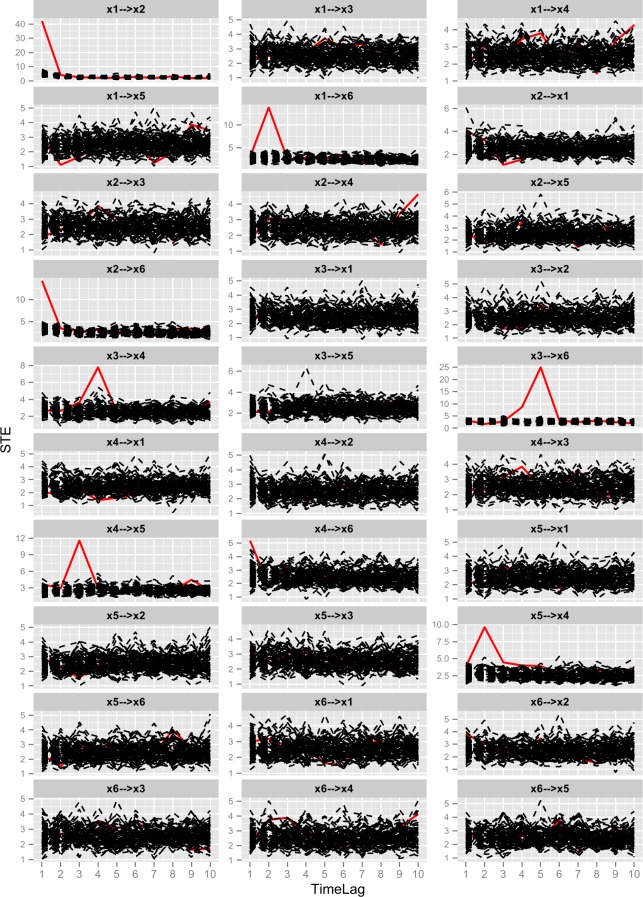
Partial symbolic transfer entropy spectrums of nonlinear system. Plot x1—>x2 is the partial symbolic transfer entropy spectrum between time series *x*_1_ and *x*_2_. Plot x1—>x3 is the partial symbolic transfer entropy spectrum between time series *x*_1_ and *x*_3_. Other plots represent the corresponding PSTES.

We apply the proposed method to this nonlinear system and the process is the same as that described in section 3.1. The resulted partial symbolic transfer entropy spectrum is shown in Fig 7. In the PSTES, if part of the red curve stands outside the other black curves, we consider the relationship between this pair of variables as a candidate strong relationship. From Fig 7, we get the candidate relationships, i.e. x1—>x2, x2—>x6, x3—>x4, x3—>x6, x4—>x5, x5—>x4, x1—>x6, x2—>x4, x4—>x6. The variable on the right of the arrow is influenced by the left one. The number of identified candidate relationships is nine pairs and the correct relationships are the first six pairs.

The candidate relationships are filtered by the method described in section 2.6.2. All the retained strong relationships are denoted as an adjacency matrix Eq (25):

$$C=\left(\begin{array}{cccccc} 0 & 1 & 0 & 0 & 0 & 0\\ 0 & 0 & 0 & 0 & 0 & 1\\ 0 & 0 & 0 & 1 & 0 & 1\\ 0 & 0 & 0 & 0 & 1 & 0\\ 0 & 0 & 0 & 1 & 0 & 0\\ 0 & 0 & 0 & 0 & 0 & 0 \end{array}\right).$$ (25)

This is a 0–1 adjacency matrix. We aim to get a weighted directed network, so we assign a weight to each edge following the method described in section 2.7. Then, we get the weighted adjacency matrix which is denoted as Eq (26):

$$C^{\prime }=\left(\begin{array}{cccccc} 0 & 42.18 & 0 & 0 & 0 & 0\\ 0 & 0 & 0 & 0 & 0 & 14.30\\ 0 & 0 & 0 & 7.77 & 0 & 24.94\\ 0 & 0 & 0 & 0 & 11.55 & 0\\ 0 & 0 & 0 & 9.60 & 0 & 0\\ 0 & 0 & 0 & 0 & 0 & 0 \end{array}\right)$$ (26)

From this matrix, we get the association network which is shown in Fig 6(B). The inferred network has six edges and they are all contained in the original network which is shown in Fig 6(B). Therefore, we consider that the proposed method works well for nonlinear system.

We also assess the performance of the proposed method when it is applied to the nonlinear system. The indicators are still precision, sensitivity [44, 45] and PTL, described in section 3.1. The results measured on ten groups of data are shown in Table 5. From Table 5, we see that the average precision of our model is higher to 0.98, the average sensitivity achieves to 0.86 and the precision of time lags identification is 0.98.

**Table 5:** The model assessment on 10 groups of data generated by Eqs (19)–(2,4). The values of Precision, Sensitivity and PTL in the table are rounded to two decimals.

ID	Precision	Sensitivity	PTL
1	1.00	0.86	1.00
2	1.00	0.86	1.00
3	1.00	0.86	1.00
4	1.00	0.86	1.00
5	1.00	0.86	1.00
6	1.00	0.86	1.00
7	1.00	0.86	1.00
8	1.00	0.86	1.00
9	1.00	0.86	0.83
10	0.86	0.86	1.00
Average	0.98	0.86	0.98

In addition, we also discuss how the dimension of symbolic time series affects the performance of the proposed method applied in nonlinear system and the results are shown in Table 6. With dimension 2, the precision is 0.92 and the sensitivity is 0.84. With dimension 3, the precision is 0.98 and the sensitivity is 0.86. For the two different parameters, the proposed method works well, especially let the dimension of symbolic time series be 3.

**Table 6:** The model assessment on different dimensions of symbolic time series from nonlinear system. The values of Precision and Sensitivity in the table are rounded to two decimals.

ID	Dimension	Precision	Sensitivity
1	2	0.92	0.84
2	3	0.98	0.86

We also discuss how the length of data affects the performance of the proposed method applied in nonlinear system and the results are shown in Table 7. It is found that the precision is always 1. The sensitivity is unstable, but it keeps a high level. Therefore, we can apply the proposed method in a small data set.

**Table 7:** The model assessment on different lengths of data from nonlinear system. The values of Precision and Sensitivity in the table are rounded to two decimals.

ID	DataLength	Precision	Sensitivity
1	500	1.00	0.80
2	1000	1.00	0.60
3	2000	1.00	0.60
4	5000	1.00	0.80

At the end of this section, we make a comparison between the proposed method and three other common methods. The results are shown in Table 8. Each value is an average value of ten-times experiments. The precision of SSPSTES is highest, i.e. 0.98. The sensitivity of SSPSTES is 0.87 and it is higher than two other methods, i.e. STE and PSTE. The sensitivity of GC is 0.98 and it is highest. But the precision of GC is lowest. Therefore, we conclude that SSPSTES is a good method for inferring association network from linear time series. The parameters and process of experiments are same as section 3.1.

**Table 8:** The performances of different methods on the same data from nonlinear system. The values of Precision and Sensitivity in the table are rounded to two decimals.

ID	Method	Precision	Sensitivity
1	GC	0.53	0.98
2	STE	0.85	0.80
3	PSTE	0.79	0.68
4	SSPSTES	0.98	0.87

### Application

**Fig 8:**
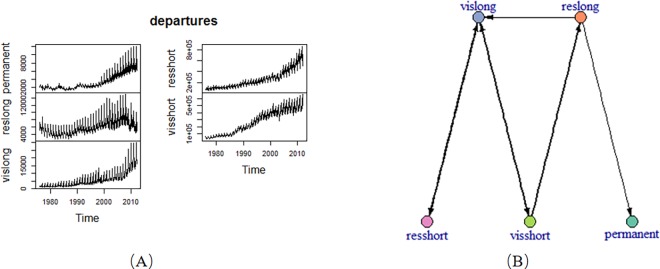
Departures time series and the corresponding inferred network. (A) departures time series; (B) the inferred network from departures data.

In this section, we apply the proposed method to a real data set, i.e. overseas departures from Australia(S3 Dataset). This data set was observed from January 1976 to February 2012. The data set has 5 time series and 434 observed point. The five time-vary features are permanent, reslong, vislong, resshort and visshort. They mean that permanent departures, long-term (more than one year) residents departing, long-term (more than one year) visitors departing, short-term (less than one year) residents departing and short-term (less than one year) visitors departing. The five time series are shown in Fig 8(A).

Based on the experiments from simulated numerical examples in section 3.1 and 3.2, we apply the proposed method to departures data set. The inferred association network is shown in Fig (B).

From Fig 8(B), we see the following pair-wise relationships. Feature vislong is influenced by reslong. They are all long-term departures. As the increase of long-term residents departing, more long-term visitors departing will happen. It is reasonable and because people look forward to go to a better place for studying, work or tour and so on. It is obvious that permanent departures will be influenced by long-term residents. In addition, feature resshort and feature visshort belong a same class. First, they are both short-term departing. Second, the relationships between them and feature vislong are both bidirectional. Of course, this conclusion is reasonable.

## Conclusions

In order to infer a weighted directed association network from multivariate time series, we have proposed a method named small-shuffle partial symbolic transfer entropy spectrum(SSPSTES) which synthesizes Symbolic Transfer Entropy(STE) and Small-Shuffle Surrogate(SSS) method and a filter algorithm. We first proposed the framework of the method. It is composed of three layers, i.e. Data Layer, Model Layer and Network Layer. Then we described the seven main process of SSPSTES from section 2.2 to section 2.7. Next, we applied the proposed method to numerical simulated linear system and nonlinear system. We used three indicators, i.e. precision, sensitivity and PTL, to assess the proposed method. We discussed how the different dimension of symbolic time series and different length of the data affect the performance of the proposed method. We also made a comparison between SSPSTES and three other relevant methods. As a result, the proposed method makes a better performance both on linear system and nonlinear system than other methods. In general, the method can identify the strong correlation and also find out the time delay between pairwise time series. Finally, we applied the proposed method to a real multivariate time series data set, i.e. overseas departures from Australia. The inferred association network is reasonable.

Although it is illustrated that the proposed method is good at inferring association network from multivariate time series, there are still some topics that are worth studying in future. First, in this paper, it is considered that the misidentification of relationships may bring with the serious consequences, thus we aim to the strong correlation identification and ignore the proportion of identified relationships among all relationships existing in the complex system. The sensitivity is unstable and sometimes may be a little low. Therefore, we will attempt to improve the sensitivity of SSPSTES. Second, the proposed method can be optimized to reduce the complexity. Third, we will apply the method to some lager systems and real complex systems, e.g. the gas pipe monitoring system and electric power monitoring system. All these topics are interesting and worth deeply studying. Nevertheless, the proposed method still can serve as a heuristic tool for inferring association network from multivariate time series so as studying the system deeply with complex network knowledge.

## Supporting Information

S1 Dataset Data from Linear System. (CSV)

S2 Dataset Data from Nonlinear System. (CSV)

S3 Dataset Application Data: Overseas departures from Australia. (CSV)
